# Multiparametric quantitative MRI of healthy adult pancreas: correlations with gender and age

**DOI:** 10.3389/fgstr.2024.1426687

**Published:** 2024-10-22

**Authors:** Lixia Wang, Lu Liang, Jiyang Zhang, Chaowei Wu, Yang Zhou, Yang Yu, Chen Zhang, Christie Y. Jeon, Tao Jiang, Srinivas Gaddam, Yibin Xie, Stephen J. Pandol, Qi Yang, Debiao Li

**Affiliations:** ^1^ Biomedical Imaging Research Institute, Cedars-Sinai Medical Center, Los Angeles, CA, United States; ^2^ Radiologic Department, Beijing Chaoyang Hospital, Capital Medical University, Beijing, China; ^3^ Department of Bioengineering, University of California, Los Angeles, CA, United States; ^4^ MR Scientific Marketing, Siemens Healthineers Ltd, Beijing, China; ^5^ Department of Biomedical Sciences, Cedars-Sinai Medical Center, Los Angeles, CA, United States; ^6^ Karsh Division of Gastroenterology and Hepatology, Department of Medicine, Cedars-Sinai Medical Center, Los Angeles, CA, United States; ^7^ Division of Digestive and Liver Diseases, Cedars-Sinai Medical Center, Los Angeles, CA, United States

**Keywords:** pancreas, magnetic resonance imaging, quantitative parameters, T1 value, T2 value, apparent diffusion coefficient

## Abstract

**Background:**

The pancreas plays an important role in the nutrition and metabolism of the whole body. Many disease processes including obesity, diabetes mellitus (DM), acute or chronic pancreatitis, and pancreatic carcinoma result in abnormality of pancreas morphology and function. Magnetic resonance imaging (MRI) provides quantitative parameters including T1, T2, and apparent diffusion coefficient (ADC) values for evaluating normal and abnormal pancreas. Based on the normal range of these quantitative parameters, pancreatic abnormality could be detected early. However, the range and the relationship of T1, T2, and ADC values with gender and age groups using the same dataset have not been explored.

**Purpose:**

To establish the ranges of MRI tissue and functional parameters, including T1, T2, and ADC values, in healthy adult pancreas and their correlations with gender, subregion, and age.

**Materials and methods:**

The T1, T2, and ADC values of healthy pancreas in 86 adults were measured using a 3.0-T MRI scanner. The average T1, T2, and ADC values were obtained in the whole pancreas and subregions (head, neck, body, and tail). Their correlations with gender and age were investigated.

**Results:**

The T1, T2, and ADC values of the whole pancreas from all subjects were 870.07 ± 61.86 ms, 44.07 ± 6.14 ms, and 1.072 ± 0.212 × 10^−3^ mm^2^/s, respectively. T2 values were significantly different between genders (*P* < 0.05). No significant differences were found between subregions. The T1, T2, and ADC values differed significantly among the age groups (*P* < 0.05). The T1 value revealed a moderately positive correlation, while the T2 and ADC values displayed negative correlations with age (*r* = 0.31, −0.45, and −0.39, respectively). The combination of T1, T2, and ADC values achieved the highest AUC value and showed a significant difference compared to T1, T2, and ADC values alone in predicting age older than 45 years.

**Conclusion:**

This study established the normal ranges of T1, T2, and ADC. We found that T2 is different between men and women, and T1, T2, and ADC are age-dependent. These results could be useful for quantitative MRI of pancreatic disease.

## Introduction

The pancreas is a retroperitoneal organ with endocrine and exocrine cells, located adjacent to the stomach and duodenum. It plays a crucial role in nutrition and metabolism. The exocrine pancreas secretes various enzymes that facilitate food digestion, while the endocrine pancreas regulates blood glucose levels. Various diseases such as obesity, diabetes mellitus (DM), acute or chronic pancreatitis, and pancreatic carcinoma can result in abnormalities in pancreatic morphology and function. In addition to volume shrinkage (1) and atrophy of the focal or whole pancreas following disease processes (2, 3), local or diffuse fat replacement of the pancreatic parenchyma (4, 5) and localized patchy fibrosis are more commonly observed in older individuals. The pancreatic microstructural changes associated with aging or the presence of disease have gained increasing attention in the fields of physiology and metabolism. These changes often exhibit a strong correlation with disease and can serve as prediagnosis biomarkers (3). Therefore, accurately evaluating and quantifying the normal ranges of these biomarkers and microstructural changes constitutes a pressing clinical concern.

MRI is a unique technique compared with ultrasound (US) (6) and computed tomography (CT) (4, 7) in pancreatic qualitative and quantitative imaging, as it provides multiple parameters to investigate the pancreatic parenchyma and fat fraction, function and metabolism, main pancreatic duct and vascular information of normal pancreas, and pancreatic disease with higher tissue resolution. Conventional T1-weighted imaging (T1WI), T2-weighted imaging (T2WI), and dynamic contrast enhancement (DCE) allow measurements of signal intensity, pancreatic volume, and evaluation of pancreatic lesions with a contrast agent. However, some challenges limit clinical MRI in accessing the biological or physiological status and monitoring treatment response. Quantitative multiparametric magnetic resonance imaging (MRI) serves as a crucial tool for detecting pancreatic structural and metabolic shifts related to aging and disease, emphasizing the chemical composition and metabolism of tissues, and providing a more accurate and direct assessment of the pathological process and tumor microenvironment without ionizing radiation. These parameters include T1, T2, T2*, and apparent diffusion coefficient (ADC) values for assessing normal and abnormal pancreatic properties (8, 9). Magnetization transfer ratio (MTR) and pH values, derived from chemical exchange saturation transfer (CEST), reflect tumor hypoxia and acidic microenvironments (10, 11). Additionally, pharmacokinetic parameters obtained from multitasking dynamic contrast-enhanced (DCE) sequences aid in evaluating blood supply and microvessels (12) and can be used in differentiating chronic pancreatitis and pancreatic ductal carcinoma (13).

Among the various parameters, T1, T2, and ADC values are the most easily and clinically measured without an additional contrast agent. ADC values serve as biomarkers that reflect cellular density and composition, helping differentiate between normal and abnormal pancreatic tissue for tumor diagnosis and grading. Although previous studies have reported conflicting results concerning the correlation between ADC values and aging (14, 15), T1 and T2 values have proven advantageous in characterizing tissue properties related to pancreatic acinar protein, endoplasmic reticulum, and pancreatic fibrosis (16, 17), as well as defining microstructural changes, such as fat, fibrosis, and inflammation (18). In this context, evaluating the normal ranges of these parameters, which may be related to age and gender, becomes essential. Prior research has primarily focused on assessing normal pancreatic volume (19) and establishing the range of pancreatic volume across different age groups (20). Some studies have found that the anterior–posterior (AP) diameter, lobulation, and parenchymal fat changes are significantly related to aging, but signal intensity showed no significant correlation (21). However, the ranges and relationships of T1, T2, and ADC values with gender, pancreatic region, and age using the same dataset have not been extensively investigated. We hypothesize that T1, T2, and ADC values can reflect the difference in pancreatic composition between gender and age. This study aims to establish the normal adult pancreas’ ranges for MR quantitative parameters, including T1, T2, and ADC values, and examine their correlations with gender, subregions, and age.

## Material and methods

### Study population

A total of 86 adults with normal pancreas [54 women and 32 men; mean age, 44.7 years (range, 18–90 years)] were recruited and studied during a 3-year period from June 2018 to June 2021. The inclusion criteria included no history of diabetes, acute or chronic pancreatitis, cystic lesions, benign or malignant tumors, or pancreatic surgery. All participants had normal amylase and lipase levels in the biochemical tests, as well as normal CA19-9 and blood glucose levels. The exclusion criteria involved the presence of pancreatic congenital abnormalities or deformations, dilation of the main pancreatic duct (MPD), severe fatty infiltration or fatty replacement of the pancreatic parenchyma on prior CT or MRI conventional sequences, and severe pancreatic atrophy resulting from previous pancreatitis or other pancreatic diseases which may skew the measurements. The subjects with poor image quality that influenced the measurements of T1, T2, and ADC values were also excluded from our study. The study design was approved by the ethics committee, and written informed consent was obtained from all participants.

### Conventional and quantitative MRI sequences

All participants underwent a 3.0-T MR scan (Biograph mMR and MAGNETOM Prisma; Siemens Healthcare Ltd., Erlangen, Germany) using an 18-channel body phase-array coil. Non-contrast quantitative MRI maps, including T1, T2, and ADC maps, were obtained. T1 mapping was performed using a 2D Modified Look-Locker Inversion Recovery (MOLLI) sequence with motion correction and a 3D B1-corrected variable flip angle (VFA) sequence for the entire abdomen, respectively. T2 mapping was carried out using a T2-prepared FLASH sequence, with location and slices copied from T1-MOLLI. Diffusion-weighted imaging (DWI)was executed using a single-shot echo-planar imaging (SS-EPI) pulse sequence with *b*-values of 50, 400, and 800 s/mm^2^.

Clinical MRI sequences were acquired simultaneously for diagnostic reference, including coronal Half-Fourier Acquisition Single-shot Turbo spin Echo imaging (T2 HASTE), transverse three-dimensional T1-weighted volumetric interpolated breath-hold examination (T1-VIBE), and DIXON fat saturation and transverse T2 BLADE sequences. A DCE sequence was obtained for some participants with parameters identical to those of T1-VIBE and DIXON. Detailed parameters can be found in **Supplementary Table S1**.

The image quality was evaluated by an experienced radiologist based on the image blur, artifacts, and deformation of the pancreas on T1 mapping, T2 mapping, and ADC maps. The subjects with poor image quality were excluded. Additionally, we evaluated the signal-to-noise ratio (SNR) of T1 mapping derived from T1-MOLLI and VFA.

### Region of interest selection

**Figure 1:**
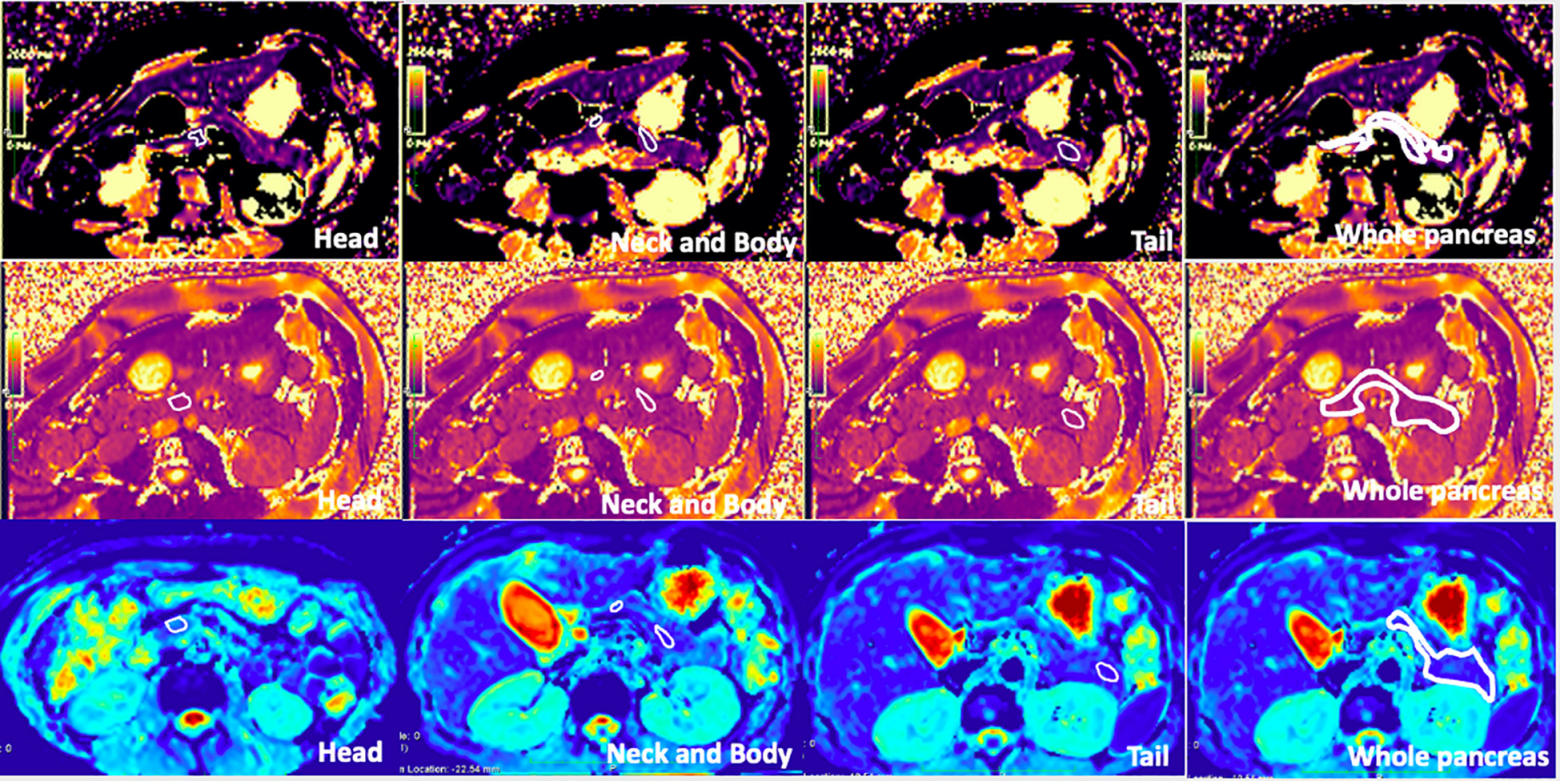
T1 mapping, T2 mapping, and apparent diffusion coefficient (ADC) map of healthy adult pancreas. Regions of interest (ROIs) of the head, neck, body, tail, and whole pancreas are shown on the images (free hand white solid line area). The T1, T2, and ADC values of the whole pancreas were 863.3 ± 30.7 ms, 46.30 ± 6.6 ms, and 1.070 ± 0.212 × 10^−3^ mm^2^/s, respectively.

Regions of interest (ROIs) were manually drawn three times in the pancreatic head, neck, body, tail, and the entire pancreas, avoiding vessels and the MPD on T1 maps by an experienced radiologist (LW). These ROIs were then copied to the T2 and ADC maps. As the ADC map is not totally matched with T1 mapping, we will first locate the ADC map using free-source Horos software (Horos v3.3.6). The average T1, T2, and ADC values for the different subregions and the whole pancreas were obtained from these three measurements. **Figure 1** shows the ROIs of different subregions and the whole pancreas.

### Statistical analysis

Quantitative values were analyzed using SPSS v25.0 software (IBM Corp, Armonk, NY, United States) and expressed as mean ± standard deviation. *t*-tests, one-way analysis of variance (ANOVA), and *post-hoc* tests (Tukey’s test) were used to compare differences among different subregions, genders, and age groups (≤30, 31–40, 41–50, 51–60, and ≥61 years). *P*-values of less than 0.05 were considered to indicate a significant difference. Pearson’s correlation coefficients were employed to investigate the correlations between age and T1, T2, and ADC values. The area under the receiver operating characteristic (ROC) curves (AUC) were performed to predict age older than 45 years.

## Results

### Demographic characteristics of the study participants

**Table 1** presents the demographic information for the 86 participants enrolled in this study, including gender, age, and ethnicity. All participants had normal blood test results for carbohydrate-associated antigen (CA19-9), alpha-fetoprotein (AFP), and carcinoembryonic antigen (CEA), as well as normal amylase and lipase in their biochemical tests within 2 weeks prior to the MRI procedure.

**Table 1:** Demographic characteristics of the study participants.

Group		Case number
Gender	Female	54
	Male	32
Age group (years)	≤30	27
	31–40	15
	41–50	6
	51–60	16
	>61	22
Ethnicity	Hispanic	12
	Non-Hispanic	67
	Do not wish to provide	7
Blood test		Mean value (normal range)
	CA19-9	10.5 U/mL (<35 U/mL)
	AFP	6 IU/mL (4.2–12 IU/mL)
	CEA	2.3 ng/mL (0–5 ng/mL)
	Amylase	65 U/L (20–125 U/L)
	Lipase	32 U/L (<60 U/L)

### T1, T2, and ADC values of the whole pancreas and the difference between gender

**Figure 2:**
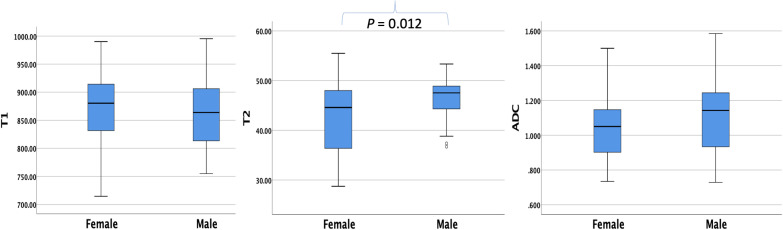
Box plots showing the T1, T2, and ADC values of healthy adult pancreas in female and male participants. The T2 value differs significantly between genders (*P* = 0.012).

The average T1, T2, and ADC values for the healthy adult pancreas were 870.07 ± 61.86 ms, 44.07 ± 6.14 ms, and 1.072 ± 0.212 × 10^−3^ mm^2^/s, respectively (**Figure 1**). **Table 2** (**Figure 2**) lists the T1, T2, and ADC values for different genders. There were no significant differences in T1 and ADC values between men and women (*P* = 0.560 and 0.298, respectively). However, T2 values were significantly different between female and male participants (*P* = 0.012).

**Table 2:** T1, T2, and ADC values of female and male pancreas.

	Gender	Mean ± standard deviation (SD)	*P*
T1 value	F	872.85 ± 58.10	0.560
	M	864.38 ± 69.56	
T2 value	F	42.67 ± 6.84	0.012
	M	46.17 ± 4.26	
ADC value	F	1.055± 0.196	0.298
	M	1.115 ± 0.234	

### T1, T2, and ADC values in pancreatic subregions and comparison according to subregions and gender

**Figure 3:**
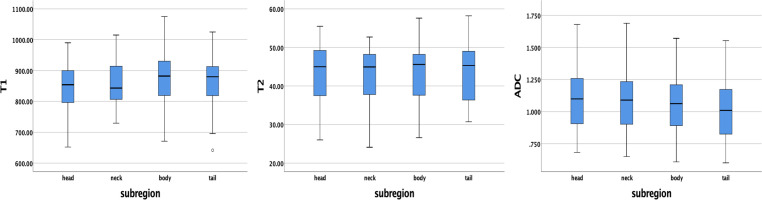
Box plots showing the T1, T2, and ADC values of healthy adult pancreas in different subregions. No significant differences were found between different subregions according to one-way analysis of variance (ANOVA).

The average ± SD of the T1, T2, and ADC values for different subregions and gender differences in each subregion are presented in **Table 3**. The T1, T2, and ADC values in the pancreatic head, neck, body, and tail were relatively consistent, with no significant differences among the various pancreatic subregions (*P* = 0.391, 0.938, and 0.456, respectively) (**Figure 3**). However, significant differences were observed in T2 values in the pancreatic neck, body, and tail between female and male participants (*P* = 0.011, 0.001, and 0.036, respectively).

**Table 3:** T1, T2, and ADC values in different subregions of normal healthy pancreas and the differences between subregions and genders.

	Subregions	Mean ± standard deviation	P*	Gender	Mean ± standard deviation	P**
T1 value	Head	858.02 ± 100.64	0.391	F	861.37 ± 104.48	0.686
				M	851.69 ± 94.66	
	Neck	871.23 ± 111.12		F	869.22 ± 92.97	0.851
				M	875.02 ±141.17	
	Body	887.72 ± 110.34		F	884.94 ± 103.23	0.773
				M	893.07 ± 124.79	
	Tail	875.81 ± 106.61		F	872.00 ± 111.12	0.702
				M	882.53 ± 111.41	
T2 value	Head	44.00 ± 7.18	0.938	F	43.25 ± 7.24	0.245
				M	45.4 ± 7.00	
	Neck	43.24 ± 7.88		F	41.35 ± 7.22	0.011
				M	46.91 ± 7.98	
	Body	43.30 ± 7.59		F	41.13 ± 7.09	0.001
				M	47.45 ± 6.88	
	Tail	43.47 ± 7.49		F	41.91 ± 7.29	0.036
				M	46.29 ± 7.17	
ADC value	Head	1.097 ± 0.266	0.456	F	1.070 ± 0.260	0.867
				M	1.163 ± 0.277	
	Neck	1.087 ± 0.259		F	1.051 ± 0.230	0.247
				M	1.169 ± 0.307	
	Body	1.068 ± 0.227		F	1.071 ± 0.216	0.169
				M	1.060 ± 0.257	
	Tail	1.026 ± 0.228		F	1.021 ± 0.224	0.805
				M	1.036 ± 0.242	

P*, differences between subregions; P**, differences between women and men for each subregion.

### T1, T2, and ADC values among age groups and comparison according to age groups and gender

**Figure 4:**
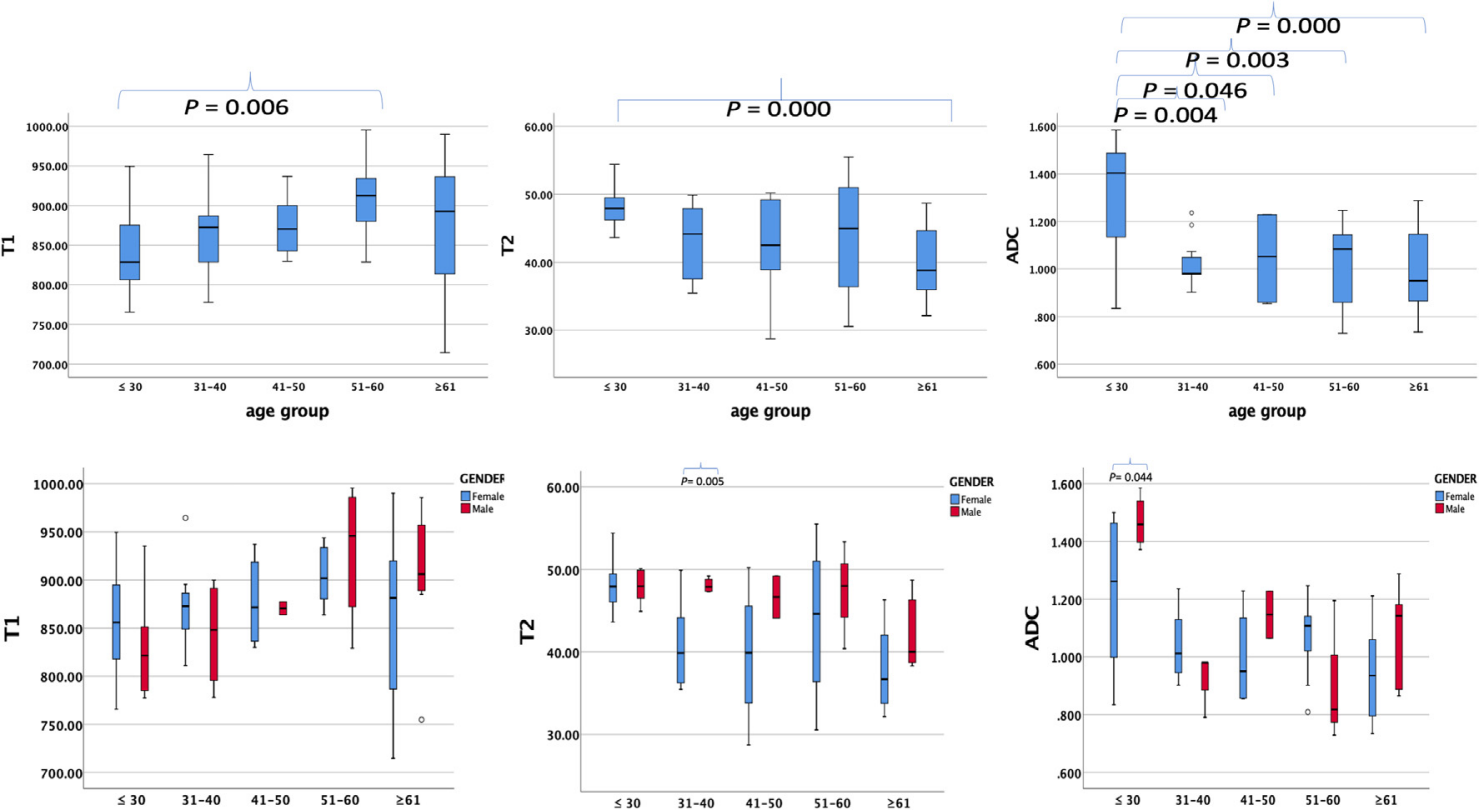
Box plots showing T1, T2, and ADC values of healthy adults’ pancreas in different age groups (upper row) and different genders (lower row) in every age group. *P*-values showing significant differences between different age groups and different genders in every age group according to ANOVA are listed in the upper box.

The average ± SD of the T1, T2, and ADC values for different age groups and genders are shown in **Tables 4**, **5**, respectively. The T1, T2, and ADC values exhibited significant differences among the age groups (*P* = 0.013, 0.001, and 0.000, respectively). The T1 value was the lowest in individuals under 30 years of age and the highest in those aged 51–60 years (*P* = 0.006). The T2 value was the highest in those under 30 years old and the lowest in those over 61 years old (*P* = 0.000). The ADC value was the highest in those under 30 years old and the lowest in those aged ≥61 years (*P* = 0.000). The *P*-values for multiple comparisons among different age groups are listed in **Table 6**, with significant differences displayed in the upper part of the box (**Figure 4**).

**Table 4:** T1, T2, and ADC values among age groups and the differences between women and men in each age group.

	Age group	Mean ± standard deviation (SD)	P*	Gender	Mean ± standard deviation (SD)	P**
T1 value	≤30	843.35 ± 52.57	0.013	F	876.65 ± 88.90	0.096
				M	824.78 ± 47.00	
	31–40	862.53 ± 47.78		F	869.44 ± 44.80	0.423
				M	843.52 ± 57.51	
	41–50	875.14 ± 39.17		F	877.48 ± 50.05	0.573
				M	870.45 ± 9.55	
	51–60	911.89 ± 44.69		F	927.82 ± 69.96	0.906
				M	929.04 ± 74.94	
	≥61	880.49 ± 83.23		F	865.89 ± 86.68	0.317
				M	905.53 ± 76.48	
T2 value	≤30	47.90 ± 2.36	0.001	F	47.91 ± 2.79	0.993
				M	47.90 ± 1.85	
	31–40	43.18 ± 5.56		F	40.73 ± 5.27	0.005
				M	52.53 ± 8.50	
	41–50	42.00 ± 7.88		F	39.67 ± 8.81	0.242
				M	46.65 ± 3.61	
	51–60	44.02 ± 8.04		F	42.74 ± 9.13	0.407
				M	46.48 ± 7.74	
	≥61	39.84 ± 5.44		F	41.03 ± 12.46	0.102
				M	39.53 ± 5.29	
ADC value	≤30	1.305 ± 0.251	0.000	F	1.223 ± 0.270	0.044
				M	1.469 ± 0.092	
	31–40	1.023 ± 0.104		F	1.039 ± 0.120	0.134
				M	0.900 ± 0.159	
	41–50	1.046 ± 0.166		F	0.996 ± 0.178	0.294
				M	1.147 ± 0.115	
	51–60	1.024 ± 0.169		F	1.081 ± 0.128	0.415
				M	0.914 ± 0.247	
	≥61	1.003 ± 0.174		F	0.909 ± 0.188	0.143
				M	1.064 ± 0.169	

P*, differences among age groups; P**, differences between women and men in every age group.

**Table 5:** The *P*-values of multiple comparisons among different age groups.

		T1 value	T2 value	ADC value
≤30	31–40	0.848	0.134	0.004
	41–50	0.752	0.152	0.046
	51–60	0.006	0.299	0.003
	≥61	0.226	0.000	0.000
31–40	41–50	0.992	0.993	0.999
	51–60	0.169	0.996	1.000
	≥61	0.901	0.527	0.998
41–50	51–60	0.703	0.948	0.999
	≥61	0.999	0.927	0.986
51–60	≥61	0.554	0.300	0.998

**Table 6:** T1, T2, and ADC values of the <45-year age group and ≥45-year age group.

	Age group	Mean ± standard deviation	P*	Gender	Mean ± standard deviation	P**
T1 value	<45	851.71 ± 51.76	0.004	F	874.30 ± 71.23	0.018
				M	829.46 ± 48.49	
	≥45	891.35 ± 67.12		F	891.79 ± 79.12	0.535
				M	907.37 ± 68.72	
T2 value	<45	46.20 ± 4.39	0.002	F	45.09 ± 5.21	0.022
				M	49.22 ± 4.87	
	≥45	41.51 ± 7.03		F	41.17 ± 10.17	0.669
				M	42.45 ± 6.35	
ADC value	<45	1.167 ± 0.231	0.004	F	1.131 ± 0.212	0.356
				M	1.308 ± 0.369	
	≥45	1.010 ± 0.168		F	0.969 ± 0.173	0.239
				M	1.043 ± 0.184	

P*, differences between age groups; P**, differences between women and men in each age group.

For comparisons of T1, T2, and ADC values between female and male participants in each age group, the *P*-values are listed in **Table 4**. The T2 value was significantly higher in men than in women aged 31–40 years (*P* = 0.005). The ADC value was significantly higher in men than in women aged ≤30 years (*P* = 0.044). However, the T1 value did not show significant differences between women and men in any age group.

When participants were divided into two age groups with a 45-year cutoff, the group aged <45 years included 44 participants, and the group aged ≥45 years included 42 participants. The T1, T2, and ADC values were significantly different between the two groups (*P* = 0.004, 0.002, and 0.004, respectively). The differences between female and male participants in the two age groups are listed in **Table 6**. The T1 and T2 values showed significant differences between female and male participants in those younger than 45 years (*P* = 0.018 and 0.022, respectively).

### Correlations of T1, T2, and ADC values with age

**Figure 5:**
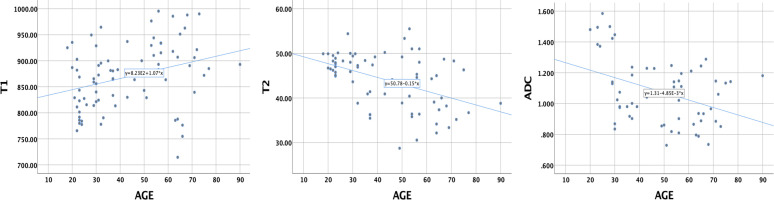
The correlations of T1, T2, and ADC values of healthy adult pancreas with age. Pearson correlation coefficients were 0.313 (*P* = 0.004), −0.453 (*P* = 0.000), and −0.394 (*P* = 0.002), respectively.

The Pearson correlation coefficients of the T1, T2, and ADC values with age were 0.313 (*P* = 0.004), −0.453 (*P* = 0.000), and −0.394 (*P* = 0.002), respectively (**Figure 5**). These results indicate that the T1 value increased with age, while the T2 and ADC values decreased with age.

**Figure 6:**
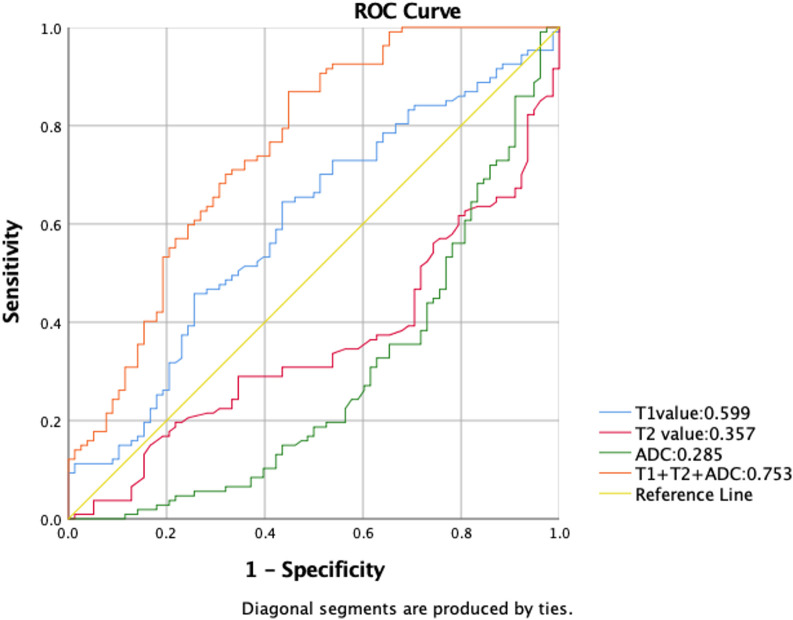
Receiver operating characteristic (ROC) curves comparing T1, T2, and ADC values used alone, as well as their combination for predicting age older than 45 years. Among the individual parameters, T1 exhibited the highest area under the curve (AUC) value of 0.599. Combining all three parameters achieved a significant improvement in AUC value of 0.753 compared to using T1, T2, and ADC values alone.

For the comparison of using T1, T2, and ADC values alone and the combination of these parameters in predicting age older than 45 years, the AUCs are shown in **Figure 6**. The combination of T1, T2, and ADC values achieved the highest AUC value and showed a significant difference compared to using T1, T2, and ADC values alone (0.753 vs. 0.599, 0.357, and 0.285).

### T1, T2, and ADC values of the whole pancreas between ethnicity

The average T1, T2, and ADC values were 867.21 ± 60.55 ms, 47.94 ± 3.23 ms, and 1.256 ± 0.209 × 10^−3^ mm^2^/s, respectively, for the Hispanic group and 861.21 ± 78.02 ms, 41.72 ± 6.80 ms, and 1.028 ± 0.218 × 10^−3^ mm^2^/s, respectively, for the non-Hispanic group. **Table 7** lists the T1, T2, and ADC values of different ethnic groups. Significant differences were demonstrated in T2 and ADC values between different ethnic groups (*P* = 0.000 and 0.000, respectively).

**Table 7:** T1, T2, and ADC values of the pancreas in different ethnic groups.

	Ethnicity	Mean ± standard deviation (SD)	P-value
T1 value	Hispanic	867.21±60.55	0.542
	Non-Hispanic	861.20±78.02	
T2 value	Hispanic	47.94±3.23	0.000
	Non-Hispanic	41.72±6.80	
ADC value	Hispanic	1.256±.209	0.000
	Non-Hispanic	1.028±.218	

### Comparison between T1-MOLLI and B1 correction VFA sequences on T1 mapping

When comparing the T1 values measured using the MOLLI and VFA techniques, there were no significant differences (854.52 ± 66.56 ms vs. 877.90 ± 91.38 ms, *P* = 0.069). However, a significant difference (*P* = 0.008) was observed in the signal-to-noise ratio (SNR = SIpancreas/SD) between the two techniques, with T1-MOLLI demonstrating a significantly higher SNR than VFA. In contrast, the contrast-to-noise ratio (CNR = SIpancreas − SImusle/SD) did not exhibit a statistically significant difference (*P* = 0.180) between the two techniques. This suggests that while the T1-MOLLI technique has a higher SNR, both techniques perform similarly in terms of CNR.

## Discussion

In summary, this study established the normal ranges of T1, T2, and ADC values for the healthy adult pancreas and investigated their correlations with gender and age. The findings showed that T2 values were gender-dependent, with higher values observed in men compared to women, while T1 and ADC values did not demonstrate a significant correlation with gender. Furthermore, the T1, T2, and ADC values were found to be age-dependent. The T1 values had a moderately positive correlation with aging, while T2 and ADC values showed moderately negative correlations with aging.

T1, T2, and ADC values have been demonstrated to effectively characterize tissue properties and distinguish between normal and abnormal tissues (22) (**Supplementary Table S2**). Previous research on myocardial tissue has revealed gender-dependent T1 values, with female patients exhibiting significantly higher T1 values than male patients. However, our study and the one conducted by Tirkes (23) found no significant differences in T1 values between the sexes. Tirkes (23) observed mild positive correlations between T1 values, extracellular volume (ECV) of the normal pancreas, and age, as the shorter T1 value properties diminished over time. However, our results demonstrated a higher Pearson correlation coefficient and measured T1 values, possibly attributable to several factors, including variances in field strength, different imaging sequences, and differences in age distribution. Herrmann (15) and Kiani Nazarlou (14) discovered that women displayed significantly higher ADC values than men for the entire pancreas (1.13 ± 0.14 × 10^−3^ mm^2^/s vs. 1.02 ± 0.18 × 10^−3^ mm^2^/s) and various regions, which was attributed to pancreatic exocrine function and fat volume. They also emphasized that variations in parameters, age groups, and body mass index values could impact ADC measurements. In contrast, our study found no significant differences in ADC values between genders, aligning with the results reported by Ma et al. (24). Our research also revealed that T2 values for the entire pancreas, as well as the pancreatic neck, body, and tail, were significantly higher in men than in women. This discrepancy may be related to increased pancreatic parenchyma volume, fat volume, fat/parenchyma ratio, and focal fibrosis in men (25). The gender-dependent nature of T2 values was different from the findings of Schwenzer (9) regarding T2* values. Discrepancies between T2 and T2* values arise because of T2 relaxation time and magnetic field inhomogeneities. Additionally, variations in field strength and the location of ROIs can influence the measured results.

The T1 and ADC values showed a slight increase and decrease, respectively, from the pancreatic head to the tail, while T2 values did not differ significantly among different anatomical regions. Vietti Violi (26) observed variation in T2 values across different anatomical locations, with the highest values noted in the tail of the pancreas. They also reported a significant correlation between T2 values and age, pancreatic main duct dilation, and diffuse pancreatic disease. In contrast, our study did not identify this trend, as we specifically selected normal pancreases based on conventional clinical MR imaging and blood test. Ding et al. (27) reported significant differences in mean ADC values among the three anatomical regions, which could be due to variations in embryological origin and cell composition (28). Specifically, the lower part of the pancreatic head and uncinate process originate from the ventral pancreas and fuse with the dorsal pancreas, while the rest of the pancreas is derived from the dorsal pancreas, resulting in differences in cell composition, tissue structure, and blood supply, leading to discrepancies in the disease spectrum. The pancreatic tail primarily accumulates islet cells, which have slower synthesis, secretion, and higher cellular density, while the pancreatic head mainly consists of acinar cells, containing rough endoplasmic reticulum and acinar proteins, leading to a lower T1 relaxation time than those observed in the other pancreatic anatomical regions, chronic pancreatitis, PDAC (29), and other abdominal organs (30). Notably, the pancreatic exocrine compartment, including the acinar and ductal epithelia, accounts for over 90% of the total pancreatic parenchyma, while the remaining fraction includes endocrine cells, blood vessels, lymphatic vessels, and other structures. Therefore, there were no significant differences in the T1, T2, and ADC values.

The study’s key finding was the correlation between T1, T2, and ADC values and age. The gradual increase in T1 values was observed due to the loss of shorter T1 properties and peaked between 51 and 60 years of age, showing a moderately positive correlation with age. However, T2 and ADC values decreased with age beyond 30 years, mainly due to alterations in fat volume, fat/parenchymal ratio (31, 32), pancreatic stiffness, structure, and function. These changes correlated with the characteristics of T2 and ADC values. Previous studies have reported reductions in total and parenchymal volumes, diffuse atrophy, and lobular atrophy with fat replacement of the pancreatic parenchyma over the decades (33). Saisho’s study (25) showed that the total pancreatic and parenchymal volumes linearly increased with age (*r* = 0.9, *P* < 0.0001) from childhood to adolescence, reached a plateau at 20–60 years, and then declined. On the other hand, the fat/parenchyma ratio gradually increased after 20 years of age and showed significant differences after 30 years of age. Our study included patients aged 18 years and above, with 75% between 20 and 60 years of age, and observed stable parenchymal volume but increased fat/parenchymal ratio. Additionally, previous studies on the elderly have reported age-related dilation of the MPD, calcification, focal fibrosis, and senile pancreatitis (21). Pancreatic exocrine function has also been evaluated in elderly individuals. However, multiple factors influenced the trends and differences in T1, T2, and ADC values. Notably, our findings differed from those of Ma et al. (15, 23, 24), who did not observe a significant correlation between ADC values and age, which could be attributed to the relatively low *b*-value in their study, leading to different ADC values.

In participants younger than 30 years, men had significantly higher ADC values than women, possibly due to differences in the integrity of the pancreatic microstructure and cell density. However, when the participants were regrouped with a 45-year cutoff, the T1 value was significantly prolonged, and the T2 and ADC values significantly decreased after 45 years of age. Furthermore, significant gender differences were observed in individuals younger than 45 years, as men exhibited greater fluid secretion due to higher levels of gender hormones. Pancreatic exocrine function is also sensitive to alcohol consumption and smoking in men (34). The gradual loss of the T1 relaxation property, diffuse and patchy pancreatic fibrosis, and changes in fluid quantification contribute to the differences between gender and age groups. When combining T1, T2, and ADC values, we observed the highest predictive performance for ages older than 45 years compared to using T1, T2, or ADC values individually. These results indicate that combining three parameters may be more accurate in identifying age-related changes in tissue characteristics within the target population. This has the potential to provide more reliable diagnostic assessments in clinical practice; aids in risk stratification and early detection of age-related pathologies; leads to more personalized and effective interventions, particularly in age-dependent conditions; and leads to a better understanding of the biological mechanisms underlying aging and age-related diseases. Tirkes (35) observed slightly lower precision in VFA measurements compared to MOLLI in a 1.5-T MR scanner, with significant differences in measured T1 values. In our study utilizing a 3.0-T scanner, we found no significant discrepancies between T1-MOLLI and VFA measurements. However, the image quality of T1-MOLLI surpassed that of VFA.

A significant difference between Hispanic and non-Hispanic subjects was found for T2 and ADC values. Nevertheless, the imbalanced number of subjects in each group may impact the comparison. Ethnicity dependence of MR parameters needs further investigation with a larger and more balanced cohort.

However, this study has several limitations. Firstly, the sample size for each age group was small, especially for the 41–50-year group. Although the study did not fully match the number of patients across age and gender, the observed trends in T1, T2, and ADC values with age and gender were consistent with previous studies. Secondly, previous research suggests that body mass index (BMI) influences ADC values (14), which was not investigated in this study.

## Conclusions

This pilot study established the normal ranges of T1, T2, and ADC. We found that T2 is different between men and women, and T1, T2, and ADC values are age-dependent. These results could be useful for quantitative MRI of pancreatic disease.

## Data Availability

The original contributions presented in the study are included in the article **Supplementary Material**. Further inquiries can be directed to the corresponding authors.
