# Supplementary material for: Multiparametric quantitative MRI of healthy adult pancreas: correlations with gender and age
Source: Front Gastroenterol (Lausanne). 2024 Oct 22;3:1426687. doi: 10.3389/fgstr.2024.1426687 (PMC12952309; doi:10.3389/fgstr.2024.1426687)
Supplement: Supplementary file 1 [file Table1.docx]

Supplementary Material

**Multiparametric Quantitative MRI of Healthy Adult Pancreas: Correlations with Gender and Age**

Lixia Wang^1^, Lu Liang^2^, Jiyang Zhang^2^, Chaowei Wu^1,3^, Yang Zhou^2^, Yang Yu^2^, Chen Zhang^4^, Christie Y Jeon^5^, Tao Jiang^2^, Srinivas Gaddam^6^, Yibin Xie^1^, Stephen J Pandol^7^, Qi Yang^2^, Debiao Li^1,3^*

*** Correspondence:**

Debiao Li

Email address: [Debiao.Li@cshs.org](mailto:Debiao.Li@cshs.org)

# Supplementary Tables

Table S1. The detailed acquisition parameters of the protocol

| parameters | T1-MOLLI | VFA | T2-prepared FLASH | DWI | T2 HASTE | T1- DIXON | T2WI blade |
| --- | --- | --- | --- | --- | --- | --- | --- |
| Slice thickness（mm） | 5 | 4 | 5 | 5 | 5 | 3 | 6 |
| Gap (mm) | 1 | 0 | 1 | 1.1 | 6 | 0 | 1.8 |
| Repetition time (ms) | 2.7 | 5.01 | 256 | 4300 | 1000 | 4.3 | 5575 |
| Echo time (ms) | 1.12 | 2.3/3.69 | 1.4 | 48 | 96 | 1.2/2.5 | 112 |
| Simulated R-R interval (ms） | 1000 |  | 1000 |  |  |  |  |
| Echo train length | 1 | 1 | 1 | 55 | 123 | 2 | 25 |
| Acquisition matrix | 192×144 | 224×135 | 192×144 | 128×84 | 256×256 | 320×195 | 320×320 |
| Flip angle | 35° | 3°, 15° | 12° | 90° | 116 | 9° | 120° |
| FOV (mm^2^) | 390×390 | 305×379 | 280×210 | 250×380 | 380×380 | 310×250 | 400×400 |
| iPAT accelarator | 2 | 2 | 2 | 2 | 2 | 2 | 2 |

**
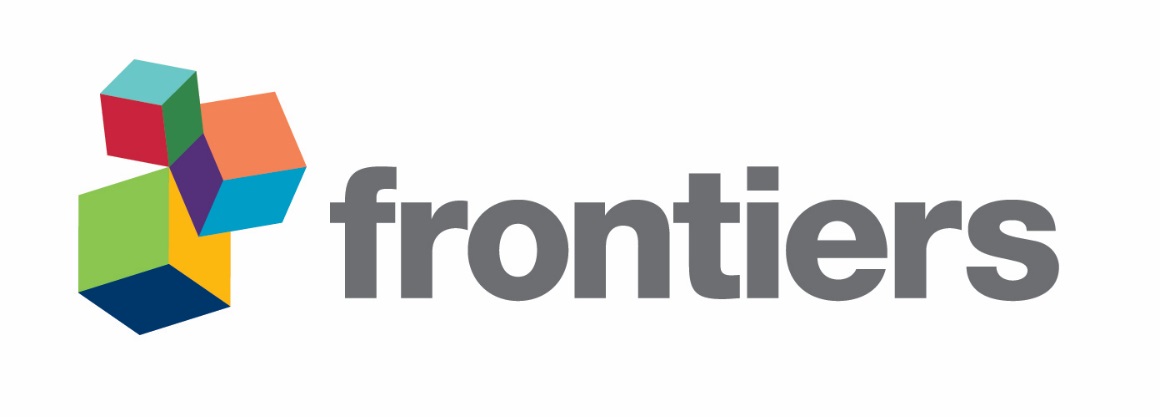
**
