# Supplementary material for: Multiparametric quantitative MRI of healthy adult pancreas: correlations with gender and age
Source: Front Gastroenterol (Lausanne). 2024 Oct 22;3:1426687. doi: 10.3389/fgstr.2024.1426687 (PMC12952309; doi:10.3389/fgstr.2024.1426687)
Supplement: Supplementary file 2 [file Table2.docx]

Supplementary Material

**Multiparametric Quantitative MRI of Healthy Adult Pancreas: Correlations with Gender and Age**

Lixia Wang^1^, Lu Liang^2^, Jiyang Zhang^2^, Chaowei Wu^1,3^, Yang Zhou^2^, Yang Yu^2^, Chen Zhang^4^, Christie Y Jeon^5^, Tao Jiang^2^, Srinivas Gaddam^6^, Yibin Xie^1^, Stephen J Pandol^7^, Qi Yang^2^, Debiao Li^1,3^*

*** Correspondence:**

Debiao Li

Email address: [Debiao.Li@cshs.org](mailto:Debiao.Li@cshs.org)

# Supplementary Tables

Table S2. The physical properties associated with each of the measures and factors affecting the measurements.

| Parameters | Technique | Physical properties | Factors affecting the measurement |
| --- | --- | --- | --- |
| T1 value | T1 mapping derived from T1-MOLLI or VFA | Spin-lattice relaxation time or longitudinal relaxation time, reflect quantification of extracellular fluid, fat/parenchymal ratio | Specific tissue (type of nuclei, the mobility of nuclear species and present of macromolecules)  Magnetic field strength (T1 time increases with field strength)  temperature  Presence of paramagnetic ions/molecules |
| T2 value | T2 mapping derived from T2 trueFISP | Transverse magnetization progressive dephasing of spin resulting in decay of the transverse plane  Occurs due to tissue characteristics which affect the movement of protons  Longest in pure water for molecular movement faster than Lamor frequency and shortest for molecular motion slower than Lamor frequency | Main magnetic field strength  Intrinsic properties of the tissue and the environment, including tissue water content, random movement of water molecules and macromolecules, Tissue fat content  Paramagnetic particles and pH value |
| ADC | ADC map derived from DWI | Microscopic changes in water moblity  Cellular density of pancreatic parenchyma | Membrane permeability  Intracellular diffusion  T2 heterogeneity  Intracellular volume fraction  Microcirculation of the tissue  b-values |

**
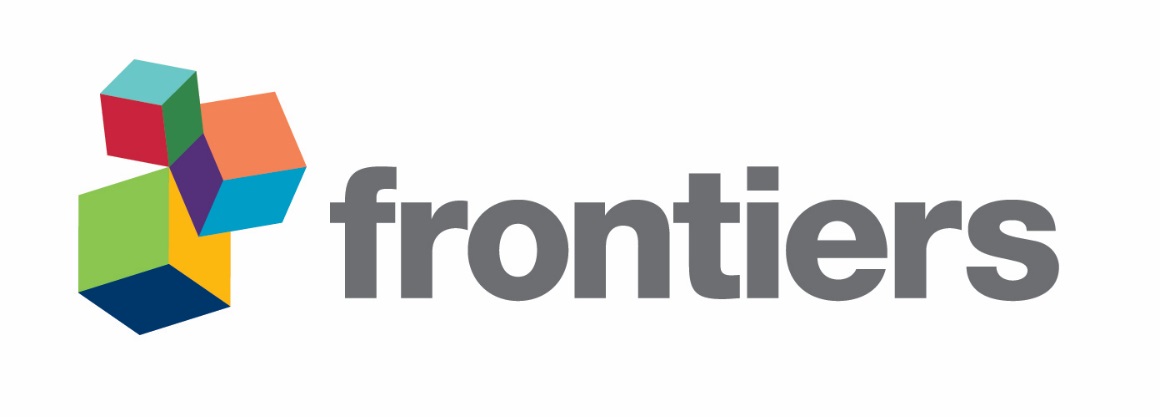
**
